# Indiana bat summer maternity distribution: effects of current and future climates

**DOI:** 10.1002/ece3.440

**Published:** 2013-01-10

**Authors:** Susan C Loeb, Eric A Winters

**Affiliations:** U.S. Department of Agriculture, Forest Service, Southern Research StationClemson, SC, 29634, USA

**Keywords:** Climate change, Indiana bats, maternity habitat, Myotis sodalis, species distribution models

## Abstract

Temperate zone bats may be more sensitive to climate change than other groups of mammals because many aspects of their ecology are closely linked to temperature. However, few studies have tried to predict the responses of bats to climate change. The Indiana bat (*Myotis sodalis*) is a federally listed endangered species that is found in the eastern United States. The northerly distribution of Indiana bat summer maternity colonies relative to their winter distributions suggests that warmer climates may result in a shift in their summer distribution. Our objectives were to determine the climatic factors associated with Indiana bat maternity range and forecast changes in the amount and distribution of the range under future climates. We used Maxent to model the suitable climatic habitat of Indiana bats under current conditions and four future climate forecasts for 2021–30, 2031–40, 2041–50, and 2051–60. Average maximum temperature across the maternity season (May–August) was the most important variable in the model of current distribution of Indiana bat maternity colonies with suitability decreasing considerably above 28ºC. The areal extent of the summer maternity distribution of Indiana bats was forecasted to decline and be concentrated in the northeastern United States and Appalachian Mountains; the western part of the current maternity range (Missouri, Iowa, Illinois, Kentucky, Indiana, and Ohio) was forecasted to become climatically unsuitable under most future climates. Our models suggest that high temperatures may be a factor in roost-site selection at the regional scale and in the future, may also be an important variable at the microhabitat scale. When behavioral changes fail to mitigate the effects of high temperature, range shifts are likely to occur. Thus, habitat management for Indiana bat maternity colonies in the northeastern United States and Appalachian Mountains of the Southeast is critical as these areas will most likely serve as climatic refugia.

## Introduction

Global climate change is predicted to have significant impacts on the world's biodiversity including range shifts, range contractions, and extinctions (Thomas et al. 2004; Malcolm et al. 2006; Huntley et al. 2008; Milanovich et al. 2010; Pereira et al. 2010). Thus, there has been considerable emphasis in recent years on developing models of future plant and animal distributions (Wiens et al. 2009). Mammals have received less attention than other organisms, perhaps because they are more likely than other taxa to show indirect responses to climate change, such as tracking the direct responses of their habitats or prey (Berteaux and Stenseth 2006). Yet, a few studies predict that climate change will result in range shifts (Adams-Hosking et al. 2011), range contractions (McCain and Colwell 2011), and declines in diversity (Currie 2001).

Temperate zone bats may be more sensitive than many other groups of mammals to climate change because their reproductive cycles, hibernation patterns, and migration are closely linked to temperature (Racey 1982; Humphries et al. 2002; Jones et al. 2009; Newson et al. 2009). For example, parturition may be delayed in some years due to the facultative use of daily torpor in response to cool temperatures (Racey and Swift 1981; Burles et al. 2009). Conversely, it has been suggested that their use of heterothermy may make them better able to adapt to warming temperatures (Boyles et al. 2011). Furthermore, many climate change scenarios forecast increasing incidences of drought or extreme weather events that may affect bat reproduction and survival (Jones et al. 2009). Most insectivorous bats must drink to maintain water balance, and water needs increase considerably during pregnancy and lactation (Kurta et al. 1989, 1990; Adams and Hayes 2008). Thus, severe droughts, particularly when coupled with unusually cold or hot temperatures, may have direct impacts on bat reproductive success (Bourne and Hamilton-Smith 2007; Adams 2010). Indirect impacts due to drought may also occur. For example, insect populations often decline during drought (Hawkins and Holyoak 1998) resulting in increased foraging costs and decreased annual survival for bats (Frick et al. 2010).

Climate change may also result in shifts in the distribution of both summer and winter ranges of temperate zone bats. Some species have already experienced range shifts including the northward extensions of Kuhl's pipistrelle bat (*Pipistrellus kuhlii*) in Europe (Sachanowicz et al. 2006), and the Brazilian free-tailed bat (*Tadarida brasiliensis*) and Seminole bat (*Lasiurus seminolus*) in the southeastern United States (Lee and Marsh 1978; Wilhide et al. 1998). Based on preferred hibernation temperatures, the winter distribution of little brown bats (*Myotis lucifugus*) is predicted to show a pronounced northward movement (Humphries et al. 2002), and the ranges of European bats are forecasted to show considerable shifts, with species in the Boreal Zone experiencing the greatest change and risk of extinction (Rebelo et al. 2010). Other than Humphries et al. (2002), Rebelo et al. (2010), Hughes et al. (2012), and Kalcounis-Rueppell et al. (2012), we are unaware of other attempts to predict or forecast changes in bat distribution in response to climate change.

**Figure 1:**
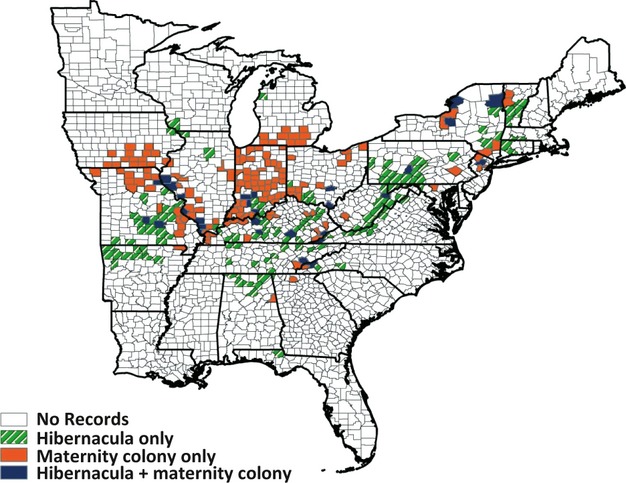
Counties within the eastern United States in which there are records for Indiana bat hibernacula, maternity colonies, and both. Source was primarily U.S. Fish and Wildlife Service (2007).

The Indiana bat (*M. sodalis*) is currently designated as an endangered species under the U.S. Endangered Species Act of 1973 (U.S. Fish and Wildlife Service 2007) and is found in parts of the northeastern, Midwestern, and southeastern United States (Fig. 1). During winter, Indiana bats hibernate in cool caves and mines in 19 states, with the most important hibernacula (≥10,000 bats) occurring primarily in the Midwest and Southeast (U.S. Fish and Wildlife Service 2007). Factors thought to have led to their decline and subsequent endangered status include destruction and degradation of hibernacula; disturbance during hibernation; and loss and degradation of summer maternity habitat, migratory habitat, and swarming sites (U.S. Fish and Wildlife Service 2007). Indiana bat populations began to increase in 2000-2005, but have declined subsequently due to White-nose Syndrome (WNS), an epizootic disease caused by a fungal pathogen (*Geomyces destructans*) that disrupts hibernation physiology and leads to death in at least six bat species (Lorch et al. 2011; Warnecke et al. 2012) including the Indiana bat (Langwig et al. 2012). Between 2006 and 2011, the number of Indiana bats hibernating in the northeastern United States declined by 72% (Turner et al. 2011).

In spring, female Indiana bats migrate ≤575 km to their summer range (Winhold and Kurta 2006) to form maternity colonies in snags and live trees of a wide range of species (Menzel et al. 2001; Kurta 2005). Although the migratory patterns of Indiana bats are not well understood, the limited data available on migration suggests that females migrate north from winter hibernacula in the Midwestern and southeastern United States (Gardner and Cook 2002; Kurta and Murray 2002; U.S. Fish and Wildlife Service 2007) and possibly short distances in a southerly direction (Britzke et al. 2006); males usually stay within the general vicinity of the hibernacula (Gardner and Cook 2002). Thus, the summer maternity range has a more northerly distribution than the winter range, particularly in the western part of the range (Fig. 1). Many studies have examined tree and microhabitat factors related to maternity roost use and have concluded that trees with greater solar exposure are likely selected because they allow passive warming of females and young, thus reducing some of the energetic costs of reproduction (Kurta et al. 1993, 2002; Callahan et al. 1997; Carter and Feldhamer 2005; Timpone et al. 2010). Yet, Indiana bats are rarely found in the warmer areas of the southeastern United States despite sufficient forested habitat (Brack et al. 2002), and when they are found in the Southeast, they are restricted to the mountainous regions (Harvey 2002; Britzke et al. 2003). This pattern suggests that on a regional or rangewide scale, climatic factors driving summer maternity range distribution may differ from those driving microhabitat selection.

Although Brack et al. (2002) conducted a qualitative examination of regional temperatures related to Indiana bat summer and winter distribution, no quantitative analyses have been conducted to determine the climatic factors related to the maternity range of the Indiana bat. The northerly distribution of Indiana bat maternity colonies relative to their winter distributions suggests that at a regional scale, Indiana bat maternity colonies prefer relatively cooler temperatures during summer. If that is the case, the warmer temperatures forecast under various climate change scenarios (IPCC 2007) may result in shifts or contractions of the area used by Indiana bat maternity colonies during summer. Thus, our objectives were to determine the climatic and topographic (i.e., elevation) factors associated with Indiana bat maternity range and forecast changes in the overall amount and distribution of the maternity range under various carbon emission scenarios and global circulation models (GCM). Because little is known about the climatic factors affecting Indiana bat maternity roost requirements, we used a correlative approach to develop a species distribution model as opposed to a mechanistic approach based on physiological and life history responses to environmental factors (Wiens et al. 2009). Although these methods have a number of underlying assumptions and uncertainties, including uncertainties in the climate models, they are a useful way to examine the potential consequences of climate change and highlight areas and species that may be most vulnerable (Lawler et al. 2009; Wiens et al. 2009) as well as areas that may serve as climatic refuges (Adams-Hosking et al. 2011).

## Materials and methods

### Study area and sample data

We included the entire eastern United States in our models (Fig. 1). Maternity records of Indiana bats from 1963 to 2007 were obtained from the draft recovery plan (U.S. Fish and Wildlife Service 2007) and additional records were obtained from the U.S. Fish and Wildlife Service. Although the records date back to 1963, 75% of the records were from 1991 or later. Because the coordinates of most maternity colonies were not available, we used the county center for each record. Multiple records within counties were not included. We used the Mean Centers Tool in ArcGIS 9.3 to determine the county centers.

### Current and future climate data

We modeled the current maternity distribution of Indiana bats using PRISM climate data for the maternity season (May–August) averaged over 1971–1999. Because our occurrence data were at the county level, we used county-level climate data. These data were based on the weighted mean averages of the underlying 5 arc minute grid (http://www.fs.fed.us/rm/data_archive/dataaccess/US_HistClimateScenarios_county_PRISM.shtml [2010, August 2]). This dataset contained the average minimum and maximum temperature for each month, the monthly precipitation totals, and elevation for each county. Because average minimum and maximum monthly temperatures for May through August were highly correlated with each other (*r* ≥ 0.95), we used maximum monthly temperature (AvgTmax) averaged across May through August. Precipitation values were not highly correlated with AvgTmax or elevation (*r* ≤ 0.70), although there was some correlation among June, July, and August monthly precipitation (0.72 < *r* < 0.82). Thus, our models contained six variables: AvgTmax (the average maximum daily temperature [ºC] for May through August), Precip5, Precip6, Precip7, Precip8 (the average monthly precipitation [mm] totals for May, June, July, and August, respectively), and Elev (m).

We used the same six variables to model future distributions of Indiana bat maternity colonies based on forecasted climates under the A1B and B2 carbon emission scenarios coupled with one of three GCMs (USDA Forest Service 2012). The A1B scenario represents a future in which there is rapid economic growth with the global population reaching a peak in the mid 21st century and then declining, and a balance between the intensive use of fossil fuels and non-fossil fuel energy sources (IPCC 2007). The B2 scenario represents a future where there is a continuously increasing global population, but at a slower rate than other scenarios; intermediate levels of economic growth; and emphasis on environmental protection and social equity. These scenarios were coupled with the CSIRO, MIROC, or Hadley GCMs (A1B-CSIRO, A1B-MIROC, B2-CSIRO, and B2-Hadley) to create a range in increasing temperatures and precipitation changes (Table 1). Models were developed for 2021–2030, 2031–2040, 2041–2050, and 2051–2060.

**Table 1:** Differences from current conditions (1971–1999) averaged over 10-year periods in mean May–August average maximum temperature (AvgTmax) and monthly precipitation for each climate change scenario/GCM combination. Standard deviations are given in parentheses

Scenario	Years	AvgTmax (ºC)	May Precip (mm)	Jun Precip (mm)	July Precip (mm)	Aug Precip (mm)
A1B CSIRO	2021–2030	1.88 (0.65)	1.68 (19.91)	−3.82 (22.38)	−11.43 (12.92)	2.37 (17.55)
	2031–2040	1.54 (0.34)	6.20 (18.59)	18.91 (19.00)	7.40 (15.31)	−2.76 (20.14)
	2041–2050	1.32 (0.48)	25.01 (24.87)	28.34 (27.61)	16.21 (22.91)	5.73 (20.53)
	2051–2060	2.88 (0.79)	6.75 (24.22)	6.28 (30.58)	−3.26 (20.91)	12.80 (21.85)
A1B MIROC	2021–2030	2.88 (0.93)	−9.75 (13.12)	−20.78 (18.37)	−15.42 (19.29)	−15.31 (18.11)
	2031–2040	3.63 (0.93)	−11.62 (1.20)	−37.16 (21.61)	−30.65 (22.10)	−24.47 (21.63)
	2041–2050	3.74 (0.99)	−10.11 (14.57)	−20.66 (19.92)	−23.85 (25.37)	−17.71 (22.68)
	2051–2060	4.93 (1.10)	−15.58 (13.98)	−32.28 (22.07)	−26.97 (20.39)	−26.42 (28.08)
B2 CSIRO	2021–2030	2.31 (0.53)	−2.79 (11.30)	−2.15 (10.10)	7.40 (18.29)	10.98 (16.88)
	2031–2040	2.57 (0.64)	−8.14 (13.93)	−0.80 (12.42)	−6.54 (16.78)	2.95 (16.32)
	2041–2050	2.64 (0.72)	−7.48 (12.11)	−6.09 (10.80)	−3.79 (18.09)	−4.82 (19.98)
	2051–2060	3.07 (0.61)	−2.38 (11.42)	−3.08 (9.55)	−5.43 (15.38)	−2.34 (15.37)
B2 Hadley	2021–2030	1.98 (0.73)	5.09 (15.23)	−6.57 (12.62)	−2.20 (13.24)	5.44 (14.48)
	2031–2040	2.76 (0.56)	−8.35 (13.62)	−10.45 (13.98)	−1.93 (14.03)	3.18 (17.29)
	2041–2050	3.36 (0.68)	1.53 (17.11)	−5.24 (18.04)	−4.71 (12.55)	0.96 (18.71)
	2051–2060	3.77 (0.93)	−1.99 (10.75)	−9.30 (13.82)	−4.55 (17.52)	−9.13 (21.01)

### Modeling procedures

We used Maxent version 3.3.3e to model current and future distributions of Indiana bat maternity colonies. Maxent models species distributions based on presence only data and performs well compared with many other ecological niche model approaches (Elith et al. 2006; Phillips et al. 2006). We created raster datasets from the county-level climate data using ArcMap 10.0 and the default raster size (0.099). We ran 20 replicates of each model where 75% of the data points were randomly selected to train the model and the remaining 25% of the sample points were used as test data in each run. We used the default settings and ran 500 iterations of each model (Phillips and Dudík 2008). The model was evaluated using a null model procedure (Raes and ter Steege 2007). We generated 1000 sets of 183 random occurrence points (equal to the number of real occurrence points in our dataset) using ENMTools Version 1.3 (Warren et al. 2010). We used Maxent to calculate the area under the receiver operating curve (AUC) for each of the 1000 null datasets and tested whether the AUC of the Indiana bat dataset exceeded the 95th percentile of the null dataset AUCs. We used the 10% training presence threshold to determine which areas were suitable for Indiana bats based on the climatic variables under current and future scenarios. We calculated the percent of the eastern United States (Fig. 1) and the percent of the current suitable distribution that were forecasted to be suitable for each time period. We also calculated the percent overlap between the current and future distributions.

## Results

### Current distribution models

**Figure 2:**
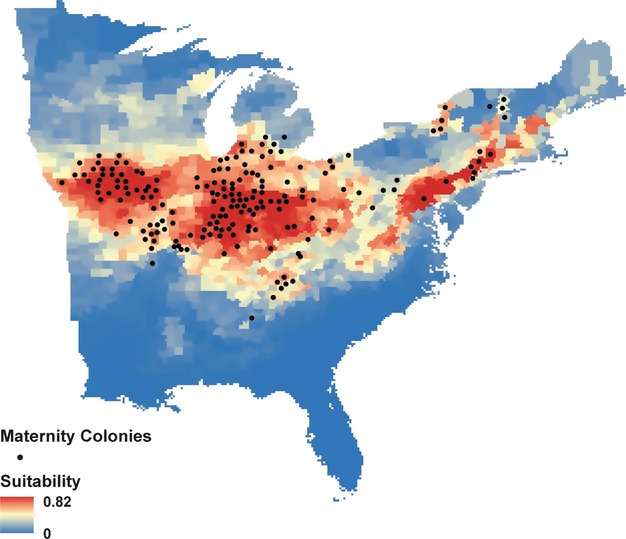
Climatic suitability for Indiana bat maternity colonies in the eastern United States based on recent (1971–1999) climatic conditions.

Approximately, 27% of the eastern United States was predicted to be suitable for Indiana bat maternity colonies based on the 1971–1999 climate and elevation data. Suitable climatic areas based on the model closely overlapped the current summer maternity range (Fig. 2). The AUC for the training dataset was 0.88 (S.D. = 0.02) and the AUC for the test dataset was 0.82 (S.D. = 0.02). The 95th percentile of the AUCs for the null training data was 0.74 and the 95th percentile of the AUCs for the null test data was 0.59. Thus, both the training and test models were significantly different from random.

**Figure 3:**
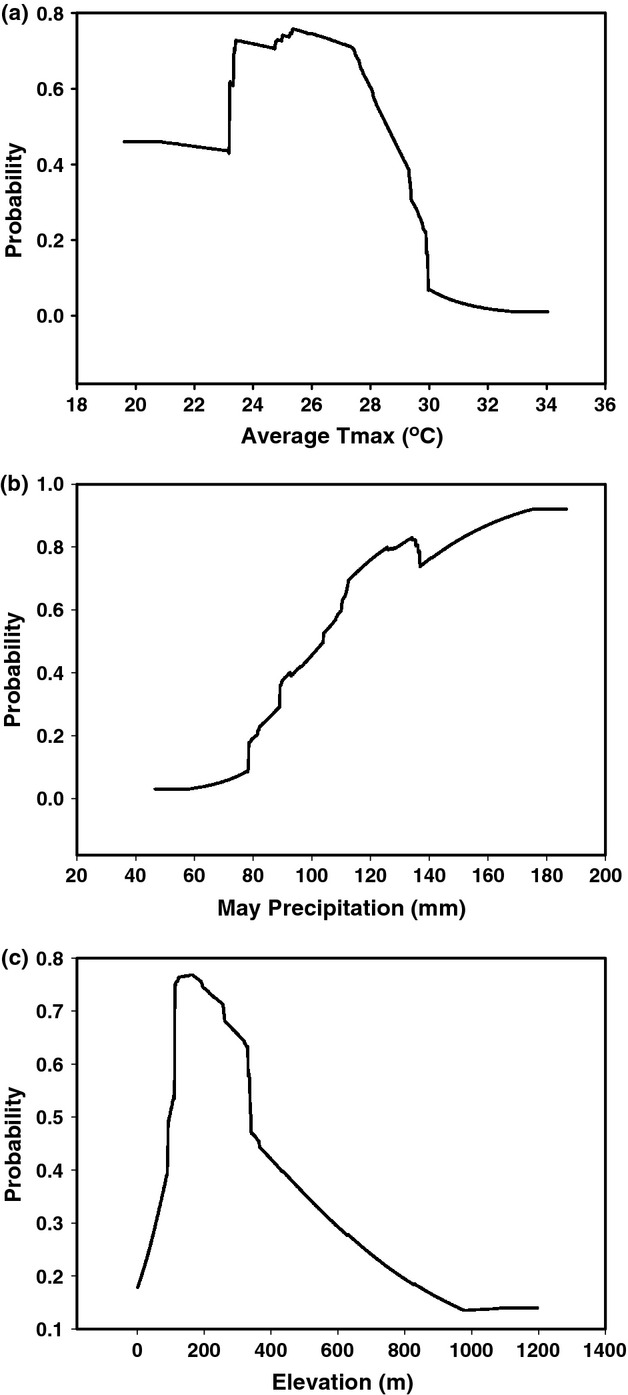
Probability that an area is climatically suitable for Indiana bat maternity colonies based on a) average maximum daily temperature from May through August, b) May precipitation, and c) elevation.

AvgTmax was the most important variable and contributed 41.5% to the model followed by Precip5 (27.6%) and Elev (24.8%). Areas with AvgTmax between 23.4ºC and 27.4ºC were most likely to be climatically suitable and the probability of presence dropped close to zero once AvgTmax exceeded 29.9ºC (Fig. 3a). The probability of maternity colony presence increased with increasing precipitation in May (Fig. 3b) while the response curve for Elev peaked at elevations between 120 m and 330 (Fig. 3c).

### Future climate scenarios and forecasts

**Figure 4:**
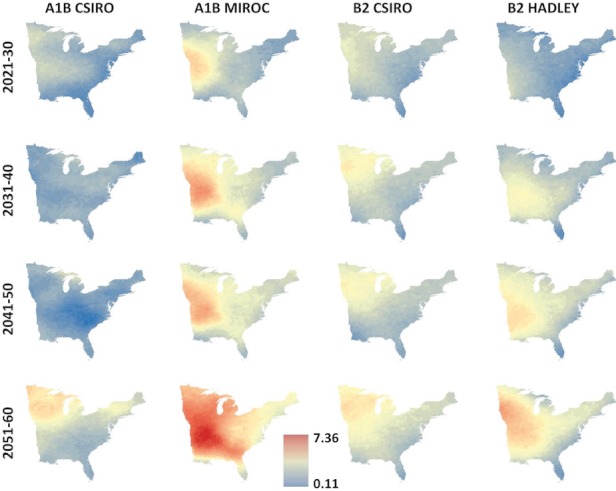
Forecasted increase in average maximum daily temperature from May through August (ºC) from recent levels (1971–1999) for four climate scenario/GCM combinations and four time periods.

AvgTmax was forecasted to increase in all climate scenario/GCM combinations between 2021 and 2060, but the temporal patterns and degree of increase varied among scenarios (Table 1, Fig. 4). Furthermore, increases in AvgTmax were not uniform across the eastern United States with the greatest increases occurring in the northern and western parts of the region (Fig. 4). Precipitation also varied with some scenario/GCM combinations forecasting increases in precipitation and others, particularly A1B-MIROC, predicting considerable declines in precipitation (Table 1, Fig. S1).

**Figure 5:**
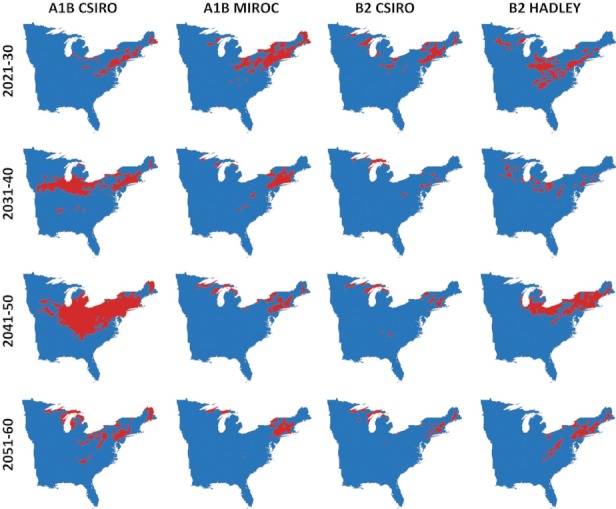
Forecasted climatically suitable areas (red) for Indiana bat maternity colonies under four climate scenario/GCM combinations and four time periods.

**Figure 6:**
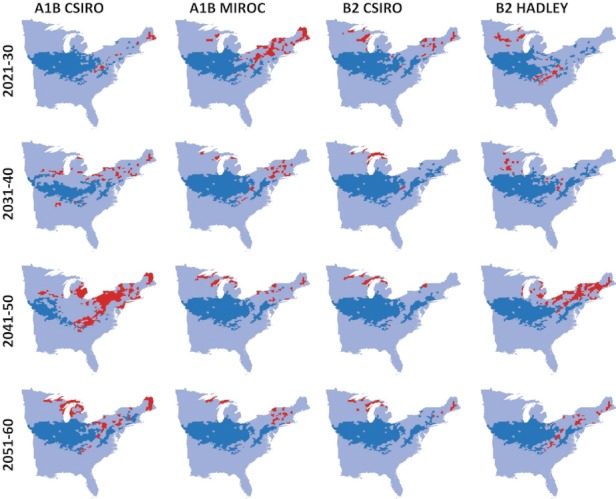
Forecasted losses (dark blue) and gains (red) in climatically suitable habitat for Indiana bat maternity colonies under four climate scenario/GCM combinations and four time periods.

Overall, the suitable area for summer maternity colonies of Indiana bats was forecasted to decline (Table 2) and be concentrated in the northeastern United States and along the Appalachian chain (Fig. 5). Most of the loss in suitable habitat was in the western and central part of the range and much of the gain in suitable habitat was in the northeastern United States, the southern Appalachians, and parts of the upper Midwest (e.g., Wisconsin and Michigan; Fig. 6). Other than the A1B-CSIRO scenario, the suitable range for Indiana bat maternity colonies was forecasted to decline to 8.3–52.8% of the current suitable range (Table 2). Under the A1B-CSIRO scenario, the suitable area was forecasted to decline to 20% of current levels in 2021–2030, but increase during 2031–2050. This increase in suitable area was due to a decrease in forecasted AvgTmax after the 2021–2030 period (Table 1 and Fig. 4) and an increase in precipitation, particularly during May (Table 1). Even though the amount of forecasted climatically suitable area in 2041–2050 was greater than the current forecasted suitable area (Table 2), much of the western portion of the current range (e.g., Missouri, Illinois, Iowa) was not forecasted to be suitable (Fig. 5 and 6). Furthermore, the amount of climatically suitable habitat was forecasted to decline precipitously again after 2050 (Table 2). The A1B-MIROC, B2-CSIRO, and B2-Hadley climate forecasts generally resulted in a decline in the amount of suitable habitat from 2021–30 to 2051–60. The amount of overlap between forecasted suitable areas and current suitable areas range from 32% to 75%. Under some future climatic conditions (e.g., A1B-CSIRO and B2-Hadley), much of the remaining climatically suitable area was within currently suitable areas, whereas in the others, much of the climatically suitable area was outside the current suitable range.

**Table 2:** Percent of eastern United States that is forecasted to be suitable for Indiana bat maternity colonies, percent of the current climatically suitable area, and percent overlap between current and forecasted suitable area under four climate scenario/GCM combinations

Climate forecast	Years	% Suitable	% of Current suitable	% Overlap with current suitable
A1B CSIRO	2021–2030	5.4	20.0	74.9
	2031–2040	15.8	58.9	75.4
	2041–2050	33.5	125.1	56.9
	2051–2060	9.8	36.6	28.7
A1B MIROC	2021–2030	11.9	44.6	45.3
	2031–2040	5.5	20.5	49.5
	2041–2050	6.1	22.7	39.8
	2051–2060	4.5	17.0	45.5
B2 CSIRO	2021–2030	6.5	24.3	53.1
	2031–2040	2.2	8.3	32.0
	2041–2050	3.3	12.4	37.0
	2051–2060	3.4	12.8	34.2
B2 Hadley	2021–2030	10.7	40.1	70.8
	2031–2040	4.1	15.3	60.9
	2041–2050	14.1	52.8	43.9
	2051–2060	5.5	20.7	54.8

## Discussion

Our analysis provides insight into the factors potentially influencing the current distribution of Indiana bat maternity colonies and suggests that the future suitable climatic range will decline over the next 50 years. We found that the most climatically suitable mean maximum temperature over the course of the maternity season was 23.4–27.4ºC. Thus, increases in AvgTmax as are forecasted under various global climate change scenarios may have profound effects on the future summer distribution of Indiana bat maternity colonies. Because most of the warming was forecasted to occur in the western portion of the current range, the heart of the Indiana bat maternity range was forecasted to shift from its current position in the Midwestern United States to the northeastern United States and the Appalachian Mountains.

Although most of the known maternity colonies fell within areas that were forecast to be highly suitable (Fig. 2), there were many areas that were predicted to be suitable that are not known to contain Indiana bat maternity colonies. Indiana bats are an endangered species and their populations are much lower than in historic times due to disturbance and destruction of hibernacula and loss and degradation of their summer habitat (U.S. Fish and Wildlife Service 2007). Thus, they have likely been extirpated from many suitable areas. Furthermore, due to their rarity, they are difficult to capture during the summer and thus, many colonies have likely not been identified. For example, maternity colonies were only recently discovered in the southern Appalachians (Britzke et al. 2003) and approximately 54% of all known colonies have been found within the past 15 years (U.S. Fish and Wildlife Service 2007). However, our models provide guidance on areas that are likely to contain maternity colonies both currently and in the future if suitable habitat exists.

Our model suggests that once average summer maximum temperatures reach 27.4ºC, the climatic suitability of an area for Indiana bat maternity colonies declines, and once AvgTmax reaches 29.9ºC, the area becomes completely unsuitable. There are few data on the response of Indiana bats to high temperatures, but one study has shown that little brown bats are better able to regulate their body temperatures at temperatures >35ºC than Indiana bats, which often succumb to the heat (Henshaw and Folk 1966). However, female Indiana bats in natural roosts in Michigan had body temperatures >35ºC on at least 1 day during the summer and several bats allowed their body temperature to exceed 35ºC on several days with one bat reaching 40.3ºC (Kurta et al. 1996). Thus, the response of Indiana bats to high temperatures needs further study, particularly in light of the results of this study.

Most studies that have examined roost-site selection at the microhabitat scale have found that Indiana bat maternity colonies select trees with greater solar exposure than random trees, likely so that they can use passive warming, particularly during pregnancy and lactation (Humphrey et al. 1977; Callahan et al. 1997; Britzke et al. 2003; Carter and Feldhamer 2005). However, selection of roosts with high solar exposure is not a universal pattern. For example, Gardner et al. (1991) found that Indiana bats in southern Illinois select roost trees in the shade and suggested that roosts in the sun would exceed the lethal temperature of Indiana bats. The Gardner et al. study is of particular note because it was conducted in the part of the current range that is forecasted to become unsuitable in all future climate scenarios. In the near future, Indiana bats may use local site selection (e.g., select trees under dense canopy) to mediate the effects of warming temperatures before abandoning an area. This may also be the case for other bat species in the eastern United States, which currently select roosts with high solar exposure such as evening bats (*Nyctieus humeralis*) and red bats (*L. borealis*, Boyles and Aubrey 2006; Perry et al. 2007). Thus, we encourage future research that examines roost-site selection and behavior of Indiana bat maternity colonies and other species in response to high temperatures as well as to low temperatures, particularly in those areas that are expected to experience larger increases in temperature over the next several decades. These studies will provide the data necessary to develop predictive models of bat responses to climate change as opposed to our forecasted models (Berteaux et al. 2006).

May precipitation also contributed to the model defining climatically suitable areas for Indiana bat maternity colonies. The probability of an area being suitable increased with increasing May precipitation. Increased precipitation in the Pacific Northwest of Canada is associated with decreased reproductive success of several *Myotis* spp. (Grindal et al. 1992; Burles et al. 2009), which may be due in part to the cool temperatures that accompany these rainy periods. In contrast, adult survival of little brown bats in the northeastern United States is positively related to precipitation during the active period (April–October; Frick et al. 2010) and reproductive success of six species of bats in Colorado declines in years of low precipitation (Adams 2010). Impacts of low precipitation may be due to decreased insect availability or inability to meet water needs during lactation (Adams and Hayes 2008). There are currently no data available on the relationship between Indiana bat demographic parameters and precipitation. However, Indiana bat maternity colonies in the Midwest and northeastern United States often select roosts close to water (Carter et al. 2002; Kurta et al. 2002; Watrous et al. 2006), presumably to reduce the flight costs to obtain water and food.

Our models forecasted a decrease in the amount of suitable maternity habitat based on climate factors for almost all scenarios and time periods. In particular, the western portion of the range (Missouri, Iowa, Illinois, Kentucky, Indiana, and Ohio), which is currently considered the heart of the Indiana bat maternity range (U.S. Fish and Wildlife Service 2007), is forecasted to become climatically unsuitable. In general, species ranges in the northern hemisphere are predicted to move northward or up in elevation in response to climate change (Parmesan 2006; Lawler et al. 2009). Although small areas in Michigan or Wisconsin were forecasted to be climatically suitable under some scenarios and time periods, the shift in forecasted suitable area was primarily eastward. This was likely due to a greater forecasted increase in AvgTmax in the western portion of the study area, even in northern areas such as Minnesota, Wisconsin, and Michigan (Fig. 4). This area was also forecasted to experience a decrease in May precipitation for many scenarios (Fig. S1). In contrast, the northeastern United States is forecasted to experience mild increases in AvgTmax. Thus, in some scenarios, the amount of suitable areas in the northeastern United States increases compared with current climatically suitable areas. The southern Appalachian Mountains were also forecasted to remain suitable under most scenarios due to mild increases in Tmax and increases in May precipitation. Because the Appalachian Mountains are topographically complex (e.g., different slopes, aspects, and landforms), they may also provide more areas that can serve as micro-refugia.

Few studies have modeled the distribution of bats in response to climate change. Rebelo et al. (2010) used similar techniques to ours to forecast species richness of European bats under four climate scenarios and found that, due to potential northward movements of species, there will likely be a change in distributions and a decline in the area occupied by bats, particularly for species in the Boreal Zone. They found that the more environmentally driven B1 and B2 scenarios resulted in fewer range contractions and fewer losses. In contrast, suitable climatic areas for Indiana bats under both the B2-CSIRO and B2-Hadley forecasts were generally lower than under the A1B-CSIRO forecasts and similar to the A1B-MIROC forecasts. Furthermore, our models did not forecast a steady decline in suitable area over time. In fact, under the A1B-CSIRO scenario, the suitable climatic area was forecasted to increase from 2031 to 2050 after a decline in the 2021–2030 period, and be greater than the current distribution during the 2041–2050 period. This is because AvgTmax was forecasted to decline slightly during 2031–2050 while May precipitation was forecasted to increase. Thus, it is important to consider the temporal patterns of climate change as well as their spatial patterns and not assume climate variables will increase or decrease linearly through time. Furthermore, when range shifts are observed, it is important to demonstrate that these range shifts correspond to changes in temperature and/or precipitation before a climate change argument is invoked. For example, although the distribution of the black flying fox (*Pteropus alecto*) in Australia has extended south over the past century, the rate of range extension far exceeds the rate of isotherm change and the species moved into colder areas than it had previously occupied (Roberts et al. 2012). Furthermore, the northward expansion of tri-colored bats (*Perimyotis subflavus*) is likely due to increased availability of hibernacula in Michigan due to anthropogenic changes and not climate change (Kurta et al. 2007).

Our models only considered climatic variables and did not include land cover or land form. Approximately, 1.8–6.5 million ha of non-federal forested land are predicted to be lost between 2010 and 2060 in the eastern United States (USDA Forest Service 2012). Thus, some areas that were forecasted to be suitable based on climatic variables may not be suitable due to lack of suitable roost habitat. However, Indiana bat maternity colonies are found in a wide variety of habitats including densely forested areas such as the southern Appalachians (Britzke et al. 2003), forested wetlands (Carter and Feldhamer 2005), agriculturally dominated areas (Humphrey et al. 1977; Callahan et al. 1997), and fragmented areas consisting of forested woodlands, agriculture, and urban and suburban areas (Belwood 2002; Sparks et al. 2005; Britzke et al. 2006; Watrous et al. 2006). Populations of Indiana bats were steady or increasing in the Midwest, Northeast, and Appalachian regions just prior to the appearance of WNS (Langwig et al. 2012; Thogmartin et al. 2012) despite increasing development and forest fragmentation in these areas, suggesting that the effects of forest loss may not be as severe for this species as it is for others (e.g., Henderson et al. 2008; Farrow and Broders 2011). However, forest fragmentation may be an important factor because Indiana bats do not fly over large open areas and restrict their foraging movements to tree lines and fence rows in fragmented areas (Murray and Kurta 2004).

It is not clear how Indiana bat maternity colonies will respond behaviorally to the change in climatically suitable habitats. Females show high multi-annual fidelity to roost areas and may migrate up to 575 km, often from different hibernacula, to reach these colonies (Kurta and Murray 2002; Winhold and Kurta 2006). Thus, initial shifts may occur at the microhabitat scale with females selecting roosts in more shaded areas than currently observed in many areas. Furthermore, disturbed areas such as gaps or those with low canopy cover may no longer be preferred. Larger scale range shifts may take more time and locating more climatically suitable areas may result in the temporary or long-term disruption of the colony structure. Climate change will likely also affect the distribution of suitable hibernacula (Humphries et al. 2002). Thus, finding suitable maternity sites may be a function of finding new hibernacula, and summer and winter range shifts may occur concurrently.

Our study suggests that maternity colonies in the western portion of the range will begin to decline and possibly disappear in the next 10–20 years. Managers must be cognizant of the potential changes in summer distributions due to climate change and not assume that declines are due to habitat loss or degradation. Colonies should be monitored closely to determine their fate and data should be collected on both changes in habitat in the surrounding area and changes in AvgTmax and precipitation. Results of this monitoring will provide important information on impacts of climate change on Indiana bats and will help predict changes in distribution beyond the next 20 years. However, declines in Indiana bat populations in the western portion of the range will likely also occur due to WNS as they have in the northeastern United States (Langwig et al. 2012). Because the northeastern United States and Appalachian Mountains are forecasted to be the most climatically suitable areas, they may serve as climatic refugia. If some colonies of Indiana bats are able to survive WNS, these climatic refugia will represent critical habitat for the species' recovery. Thus, management actions which foster high reproductive success and survival, such as providing large diameter snags in a variety of habitat types, will be critical for the conservation and recovery of the species (Menzel et al. 2001; U.S. Fish and Wildlife Service 2007).

There is much uncertainty in species distributions models such as the ones we developed for the Indiana bat. These uncertainties include the climate model projections as well the quality and extent of the data that are used to model current distributions (Wiens et al. 2009). We tried to minimize this uncertainty by using a range of future climate forecasts based on two emission scenarios coupled with several GCMs (Beaumont et al. 2008) and restricting our forecasts to the next 50 years. However, all of the models forecasted eventual declines in climatically suitable area and significant changes in the distribution of Indiana bat maternity range. Thus, the effects of climate change should be considered in future threats analyses and conservation strategies for the Indiana bat. Furthermore, managers and biologists cannot assume that because Indiana bats are not currently present on their landscape, they will not be there in the future. Thus, it is necessary to continue to survey for this species throughout its potential summer range, particularly in the northeastern United States and along the Appalachian chain. Future studies of Indiana bat roost-site selection should focus on behavioral and physiological responses to high temperature as these studies will allow more precise predictions of Indiana bats' responses to climate change.

## Supplementary material

**Figure S1**. Forecasted change in May precipitation (mm) from recent levels (1971–1999) for four climate scenario/GCM combinations and four time periods.
