# Supplementary figures and images for: Indiana bat summer maternity distribution: effects of current and future climates
Source: Ecol Evol. 2013 Jan 10;3(1):103–14. doi: 10.1002/ece3.440 (PMC3568847; doi:10.1002/ece3.440)

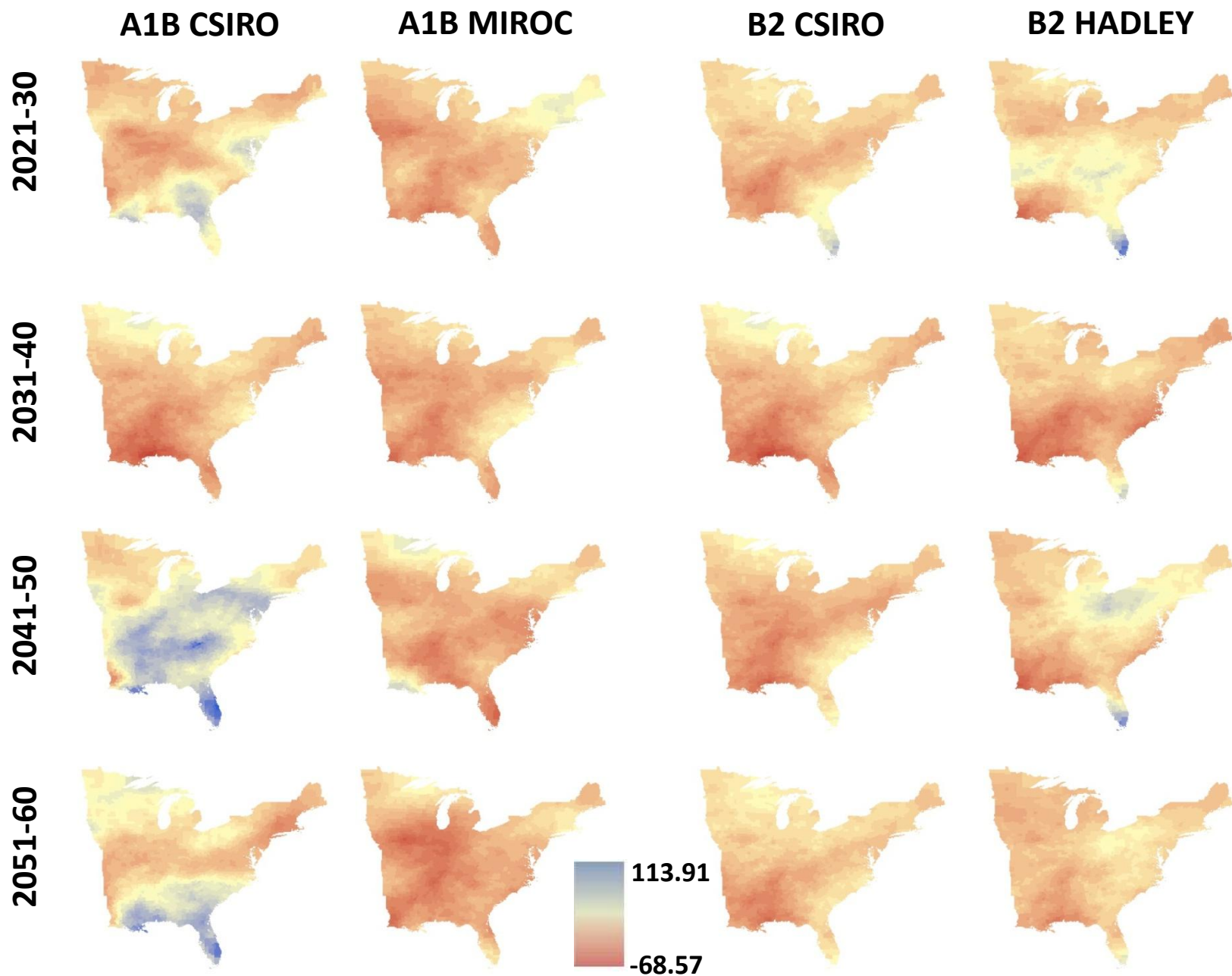

Supplement: Supplementary file 1 [file ece30003-0103-SD1.pdf]

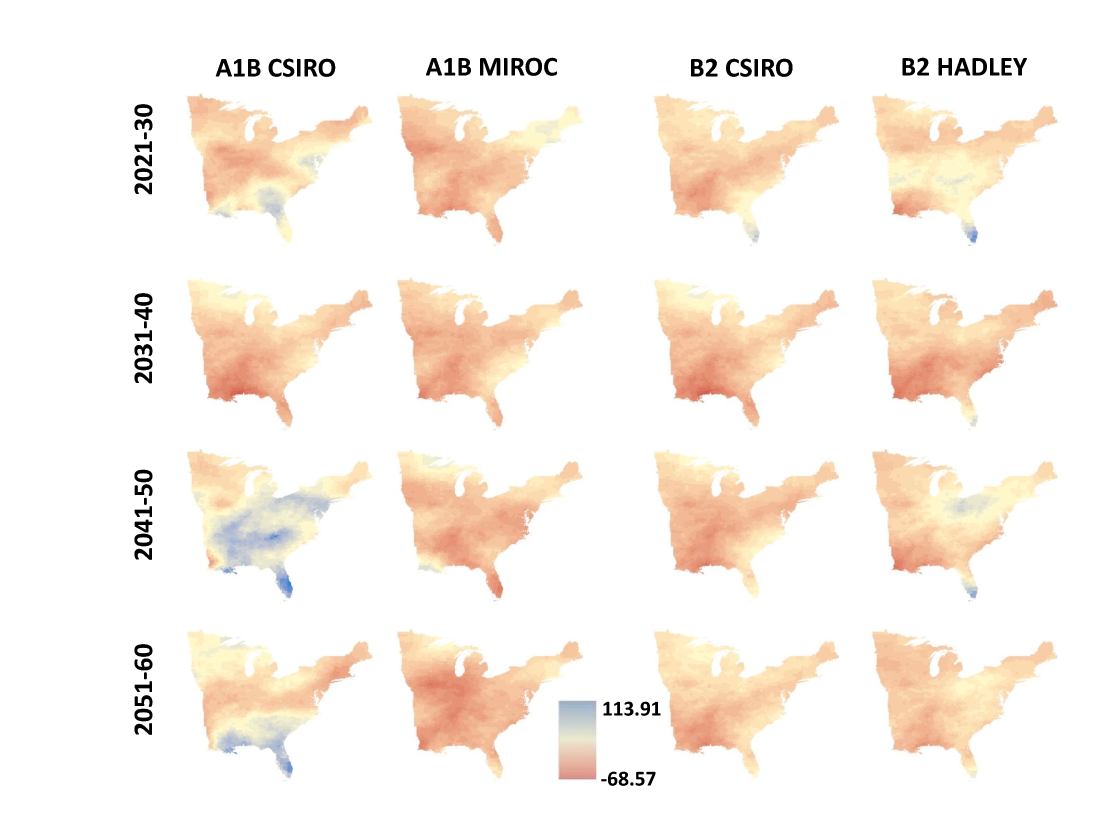

Supplement: Supplementary file 2 [file ece30003-0103-SD2.png]
